# Safety and efficacy of lumen-apposing metal stents and double-pigtail plastic stents for endoscopic ultrasound-guided drainage of walled-off necrosis; a systematic review and meta-analysis

**DOI:** 10.1080/07853890.2022.2164048

**Published:** 2023-02-13

**Authors:** Hayat Khizar, Hu Yufei, Wu Yanhua, Chen Wangyang, Bian Ying, Le Chenyu, Huang Zhicheng, Kamran Ali, Yang Jianfeng

**Affiliations:** a Department of Gastroenterology, International Education College of Zhejiang Chinese Medical University, Hangzhou, Zhejiang, China; b Department of Gastroenterology, Affiliated Hangzhou First People’s Hospital, Zhejiang University School of Medicine, Hangzhou, China; c Department of Gastroenterology, The Fourth School of Clinical Medicine, Zhejiang Chinese Medical University, Hangzhou, Zhejiang, China; d Department of Oncology, The Fourth Affiliated Hospital, International Institute of Medicine, Zhejiang University School of Medicine, Hangzhou, China; e Key Laboratory of Clinical Cancer Pharmacology and Toxicology Research of Zhejiang Province, Hangzhou, China; f Key Laboratory of Integrated Traditional Chinese and Western Medicine for Biliary and Pancreatic Diseases of Zhejiang Province, Hangzhou, China; g Hangzhou Institute of Digestive Diseases, Hangzhou, China

**Keywords:** Lumen-apposing metal stents, EUS-guided drainage, walled-off necrosis, plastic stent, meta-analysis

## Abstract

**Background:**

Patients with walled-off necrosis (WON) are still challenging to treat safely and effectively. Recently, double-pigtail plastic stents (DPS), bi-flanged metallic stents (BFMS), and lumen-apposing metal stents (LAMS) have been employed with endoscopic ultrasound-guided (EUS-guided) drainage. However, there is little solid evidence to support the effectiveness and safety of using stents. This study aims to compare the outcomes of the LAMS and the PS.

**Method:**

Till July 2022, a thorough database search was done, and studies that met the criteria were chosen. By using the RevMan software, the technical and clinical success and other secondary outcomes were calculated. Subgroup analysis was performed between the LAMS and the BFMS.

**Results:**

Fifteen studies (two randomized controlled trials and thirteen observational) with 687 patients receiving metal stents and 771 patients receiving plastic stents were selected for final analysis. There was no significant risk of bias or publication bias. The odds ratios (OR) for technical and clinical success were 0.36 (95% confidence interval (95% CI) 0.08, 1.52) and 2.26 (95%CI 1.62, 3.15), respectively. The OR for overall adverse events was 0.74 (95% CI 0.41, 1.34). In subgroup analysis, the LAMS and the BFMS showed the same outcomes.

**Conclusion:**

Compared to DPS, LAMS had better clinical outcomes and fewer side effects when treating patients with WON.

## Key Messages

- What is the best endoscopic treatment option for the walled-off necrosis (WON)?
- A brief comparison of lumen-apposing metal stents (LAMS), bi-flanged metallic stents (BFMS) and double-pigtail plastic stents (DPS) for the treatment of WON patients.
- How can we limit the adverse events and provide better treatment.

## Introduction

Pancreatic fluid collection (PFC) is a major problem for patients suffering from chronic pancreatic inflammation. Pancreatic pseudocysts (PP) and walled-off necrosis (WON) are examples of PFC that have lasted longer than 4 weeks in a patient. On the other hand, examples of pancreatic fluid that is <4 weeks old include acute peri-pancreatic and acute post-necrotic fluid collections. WON has a death rate of between 8% and 39%, which can happen to about 20% of people with severe pancreatitis [1,2]. The majority of PFCs can dissolve naturally. Abdominal discomfort, an infection, or an obstruction of the biliary or gastric tract necessitates the drainage of pancreatic fluid [3]. Therefore, a multidisciplinary approach, including surgery, radiology and endoscopy, is typically required to treat PFCs, especially WONs. Drainage of PFC utilizing transmural stents guided by endoscopic ultrasonography [4] has been the standard treatment method since 1996 [3,5,6]. For WON, a mature encapsulated collection of pancreatic necrosis that contains both liquid and solid components, transmural drainage is insufficient and endoscopic necrosectomy may be required [7–10].

EUS-guided drainage can utilize a variety of stents, including double-pigtail plastic stents (DPS), self-expanding metallic stents, and lumen-apposing metal stents (LAMS). DPS has been the therapeutic choice for endoscopic treatment of WON to access and drain PF. But these stents have several drawbacks, such as ineffective drainage, secondary infection, bleeding, perforation and the requirement of tract balloon-dilation for endoscopic necrosectomy for the endoscope to enter the collection. Additionally, these stents increase the risk of bleeding during endoscopic necrosectomy [11–15]. Since LAMS are so simple to use and offer obvious benefits, including a wide path for direct endoscopic necrosectomy and drainage, they have largely replaced traditional methods of treating PFCs [16]. Multiple studies have shown that LAMS is either more effective than DPS or is equally effective. These trials support that LAMS is effective, showing similar adverse outcomes as DPS. However, there is limited information from RCT studies, and LAMS are more expensive.

This meta-analysis was conducted to determine whether LAMS is safe and effective for draining PFC in WON patients. The studies that compared the outcomes of both WON and PP or PP only were not included in this analysis.

## Materials and methods

The Preferred Reporting Items for Systematic Reviews and Meta-Analysis guidelines were followed in this systematic review and meta-analysis [17].

### Search strategy

We searched medical databases such as PubMed, Web of Science, and the Cochrane library using various word combinations such as ‘endoscopic ultrasound-guided drainage’, ‘stents’, ‘plastic stent’, ‘lumen-apposing metal stent’, ‘PFCs’ and ‘WON’. Our search was conducted up until July 2022. The investigation was restricted to only human subjects and the English language. Two different authors were responsible for collecting all of the relevant references, and disagreements were settled through discussion.

### Studies selection

The following criteria were used to determine which studies should be included and which should be eliminated.

#### Inclusion criteria

1. Studies comparing the outcomes of metal stents and plastic stents in EUS-guided drainage.
2. Research involving only patients diagnosed with WON.
3. Full-text studies comparing technical and clinical success.
4. Human subject studies in the English language.

#### Exclusion criteria

1. Case reports, abstracts, reviews and letters were not included.
2. Studies comparing other PFCs, such as PP or a combination of PP and WON.
3. Single-arm studies such as reporting only metal stents or plastic stent outcomes.
4. Studies with incomplete results or missing technical and clinical success.
5. Articles are not published in the English language.
6. Research in the field of paediatrics.
7. Animal studies.

### Data extraction

Two writers independently collected study data based on inclusion criteria. Each study’s name, year of publication, study design, place of study, total number of patients in each arm, mean age of patients, male and female percentage, mean size of PFC, technical and clinical success, mean number of procedure sessions, mean procedure time, hospital stay, total adverse events (AEs), bleeding, perforation, stent migration and occlusion and infection were collected. Different coefficients were rescaled. Each study had LAMS/BFMS and DPS arms.

### Outcomes and definitions

Our primary outcomes were technical success (TS) and clinical success. TS is defined as successful stent installation and access to the PFC site. In contrast, clinical success is defined as a reduction in the volume of the PFC and an improvement in the clinical symptoms. The secondary outcomes were the average number of procedure sessions, mean procedure time, AEs (infection, bleeding, perforation and stent migration or occlusion), hospital stay, mortality and the requirement for surgical necrosectomy. In subgroup analysis, studies were divided into LAMS and BFMS subgroups.

### Risk of bias assessment of included studies

We used Cochrane risk of bias assessment tools for RCT studies, whereas non-RCT studies were assessed by non-RCT studies’ risk of bias assessment tools. When a study had a low risk of bias, a ‘low’ grade was assigned; ‘some concern’ for a moderate risk of bias or information reported was insufficient to make a risk of bias decision; and ‘high’ for higher risk of bias [18].

### Publication bias and study effect

Using a funnel plot, this study identified publication bias. Data on the funnel plot appeared symmetrical, indicating no publishing bias. Each study’s effect on the overall result was evaluated by first deleting each study individually and then analysing how this changed the overall result.

### Statistical analysis

We used the Cochrane Review Manager Software (version 5.4.1) to calculate odds ratios (ORs) and pooled mean differences for outcomes. We used the Mantel-Haenszel random effect model for dichotomous data to calculate OR with their 95% confidence interval (95% CI). On the other hand, we used an inverse variance statistical method and a random effect model to determine the pooled mean difference between the two groups for continuous data. For the heterogeneity calculation, chi-square tests and *I*^2^ statistics were used. *I*^2^ values between 0% and 25% were considered potentially insignificant, 25%–49% were considered lower heterogeneity, 50%–74% were regarded as moderate heterogeneity, and values over 75% were considered higher heterogeneity. A *P*-value <0.05 was considered statistically significant [19].

## Results

### Study selection

**Figure 1.**
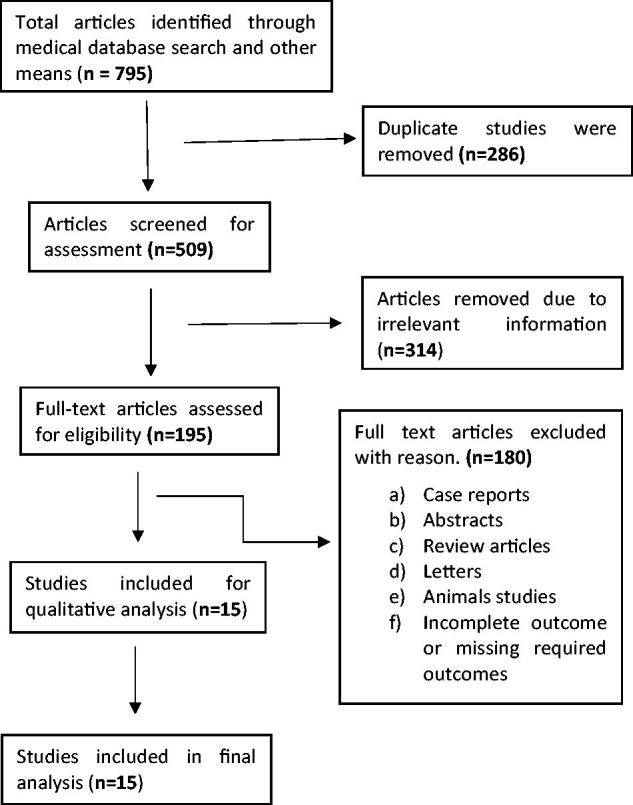
Chart for studies selection.

Medical databases and other resources yielded 795 articles. After reading topics and abstracts to remove duplicate and irrelevant articles, 195 articles were evaluated for eligibility. After removing abstracts, case reports, reviews, letters, animal studies, and incomplete or missing outcome studies, 15 studies were selected. The flow chart shows that 15 publications were chosen for the final meta-analysis (Figure 1 flow chart).

### Studies characteristics

A total of 1458 patients were included in the selected studies, of which 687 patients received metal stents (512 received LAMS and 175 received BFMS), while 771 patients underwent plastic stents. Two RCTs [20,21] and thirteen observational studies [22–34] compared the LAMS/BFMS and DPS outcomes of patients with WON. Seven studies were carried out in the USA [20,22–24,29,30,34], three in India [27,28,33] and one each in Japan [26], Saudi Arabia [25], Sweden [31], the Netherlands [21] and China [32]. Tables 1 and 2 display all study characteristics. WON aetiologies include alcohol, gallstones, idiopathic diseases, hypertriglyceridaemia, post-ERCP, trauma and drugs; 7–10 Fr DPS were used. Most study utilized HOT AXIOS (Boston Scientific, USA) LAMS, SPAXUS (Taewoong Medical, Korea), NAGI (Niti-S, Taewoong Medical CO, Korea), and Micro-Tech (Micro-Tech CO, China).

**Table 1.** Characteristics of included studies.

Name of study	Study design/country	No of patients	F/M% age	Mean age	Mean size of collection	TS	Clinical success	Mean number of procedure	Mean procedure time (min)
Mukai et al. 2014 [26]	OS Japan	BFMS 43	6/37	54.4	105 mm	43/43	42/43	2.7 ± 1.8	79 ± 80
		DPS 27	6/21	55.9	77.1 mm	27/27	25/27	4.1 ± 3.4	97 ± 117
Siddiqui et al. 2016 [29]	OS USA	LAMS 86	9/77	51.5	113.7 mm	84/86	77/86	2.2	50.4
		DPS 106	38/68	56.3	106 mm	105/106	86/106	3.6	56
Bang et al. 2016 [22]	OS USA	LAMS 13	N/A	50.7	120 mm	13/13	12/13	N/A	9.2 ± 4
		DPS 26		52.9	109 mm	26/26	24/26		33.8 ± 24
Abu Dayyeh et al. 2018 [23]	OS USA	LAMS 58	13/45	52.7	134 mm	58/58	55/58	1.1 ± 1.6	N/A
		DPS 36	8/28	59.7	128 mm	36/36	33/36	1.7 ± 1.9	
Sahar et al. 2017 [30]	OS USA	LAMS 25	8/17	51.2	153 mm	25/25	22/25	N/A	N/A
		DPS 25	11/14	53.2	137 mm	25/25	24/25		
Bapaye et al. 2017 [33]	OS India	BFMS 72	62/10	43.8	100.9 mm	72/72	68/72	1.98 ± 0.5	N/A
		DPS 61	54/7	40.6	117.1 mm	61/61	45/61	2.62 ± 0.75	
Bang et al. 2018 [20]	RCT USA	LAMS 31	11/20	55.8	102 mm	31/31	29/31	2.8 ± 1.2	18 ± 15.5
		DPS 29	13/16	60.3	107 mm	29/29	28/29	3.2 ± 1.5	41.6 ± 25.7
Chen et al. 2019 [24]	OS USA	LAMS102	54/48	54	111.4 mm	102/102	82/102	2.6 ± 1.5	N/A
		DPS 87	41/46	57	135 mm	86/87	50/87	3.1 ± 1.5	
Rana et al. 2020 [28]	OS India	LAMS 28	6/22	39.2	119 mm	27/28	27/28	3.33	N/A
		DPS 138	22/116	37.1	106 mm	138/138	136/138	3.53	
Zhu et al. 2020 [32]	OS China	LAMS 54	15/39	45.4	108 mm	54/54	48/54	N/A	N/A
		DPS 30	11/19	48.1	106 mm	30/30	21/30		
Lehibi et al. 2020 [25]	OS S. Arab	LAMS 13	N/A	43.5	N/A	13/13	13/13	N/A	N/A
		DPS 2		43.5		2/2	1/2		
Ge et al. 2020 [34]	OS USA	LAMS 34	13/21	52.5	84.7 mm	31/34	30/34	1.5 ± 0.8	N/A
		DPS 78	30/38	51.9	92.7 mm	78/78	60/78	1.5 ± 0.8	
Muktesh et al. 2022 [27]	OS India	BFMS 60	N/A	41.1	N/A	59/60	57/60	N/A	16.8 ± 4.6
		DPS 60		38.6		59/60	51/60		43.27 ± 9.6
Boxhoorn et al. 2022 [21]	RCT Nether.	LAMS 53	20/33	59	N/A	53/53	34/53	2.5 ± 2.17	N/A
		DPS 51	17/34	63		51/51	27/51	2.5 ± 1.77	
Valente et al. 2022 [31]	OS Sweden	LAMS 15	4/11	59.9	131 mm	15/15	14/15	4.8	N/A
		DPS 15	4/11	49.8	124 mm	15/15	13/15	1.5	

DPS: double-pigtail plastic stents; LAMS: lumen apposing metal stent; N/A: no information; OS: observational study; RCT: randomized controlled trail; WON: walled-off necrosis.

**Table 2.** Characteristics of included studies.

Name of study	Hospital stay	Total AE	Bleeding	Perforation	Mortality	Stent Mig/Occu	Recurof WON	Surg.NS	Additional Percut. drainage	Infection
Mukai et al. [26]	22.5 ± 10.1	3	0	1	0	2	N/A	N/A	N/A	N/A
	28.7 ± 17.9	5	3	0	2	1				
Siddiqui et al. [29]	N/A	11	6	3	0	0/3	N/A	8	N/A	1
		14	2	1	3	3/23		20		5
Bang et al. [22]	13.5 ± 33.4	4	N/A	N/A	N/A	2	N/A	N/A	N/A	N/A
						1				
	12.5 ± 22.3	3								
Abu Dayyeh et al. [23]	5 ± 2.6	21	4	1	1	12/2	N/A	N/A	N/A	2
	12.4 ± 5.4	20	7	3	2	7/1				2
Sahar et al. [30]	21.8 ± 14.2	6	1	N/A	N/A	1	N/A	N/A	N/A	4
	18.25 ± 11.	8	3			1				5
Bapaye et al. [33]	4.1 ± 2.6	4	2	N/A	3	2	N/A	2	N/A	2
										16
	8 ± 5.3	22	5		4	2		16		
Bang et al. [20]	6.2 ± 9.0	13	3	0	1	2	1	1	3	N/A
	12.2 ± 21.1	6	0	0	1	2	0	0	2	
Chen et al. [24]	N/A	10	N/A	N/A	N/A	3/21	5	5	N/A	N/A
		9				6/11	20	14		
Rana et al. [28]	N/A	2	2	N/A	1	N/A	N/A	0	N/A	0
		10	4		9			9		
										2
Zhu et al. [32]	N/A	13	3	N/A	N/A	2/2	2	N/A	N/A	7
		19	2			5/3	2			8
Lehibi et al. [25]	N/A	1	N/A	N/A	N/A	1	N/A	N/A	N/A	0
		1				0				1
Ge et al. [34]	N/A	14	3	3	1	1/4	2	0	1	1
		6	1	0	0	1/2	18	10	5	1
Muktesh et al. [27]	5.5 ± 9.8	4	1	0	0	2	1	1	0	N/A
	8.5 ± 7.8	8	4	1	2	1	2	0	4	
Boxhoorn et al. [21]	43 ± 32.6	14	9	1	6	N/A	N/A	N/A	17	N/A
									14	
	53 ± 39.11	20	11	4	9					
Valente et al. 2022 [31]	91.3 ± 21.5	6	2	0	0	2	N/A	1	N/A	2
										3
	18.45 ± 7.3	5	0	0	1	1		2		

AE: adverse events; Mig/Occu: migration/occlusion; Percut: percutaneous; Surg. NS: surgical necrosectomy.

### Risk of bias and Publication bias

Two RCTs were low-risk, while 13 observational studies were moderate-risk (Table S1). All studies and outcomes had moderate bias. A funnel plot of outcome data was used to assess publication bias. Our study found symmetric funnel plots. Each study’s impact on the outcome was examined by eliminating it. All the studies had little effect, and the ultimate result was unaffected (supplementary Funnel plots). If a study’s outcome changed significantly, we would exclude it from the analysis.

## Primary outcomes

### Technical success

Ten out of fifteen studies reported 100% TS for both groups (as shown in the Table 1 TS column). Only five studies reported different TS and were utilized to calculate the TS OR between LAMS and DPS. The OR of TS was 0.36 (95% CI 0.08–1.52), *I*^2^ = 20% and *P* = 0.16. This shows that OR of TS for LAMS and DPS was not significantly different with no heterogeneity between the two groups (Figure 2a). That means TS is almost same for both LAMS and DPS. This difference may be related to difficulty to implant the stent due to different location and anatomy of the patients.

Figure 2.Forest plot for (a) TS, (b) clinical success and (c) total AEs.

**Figure:**
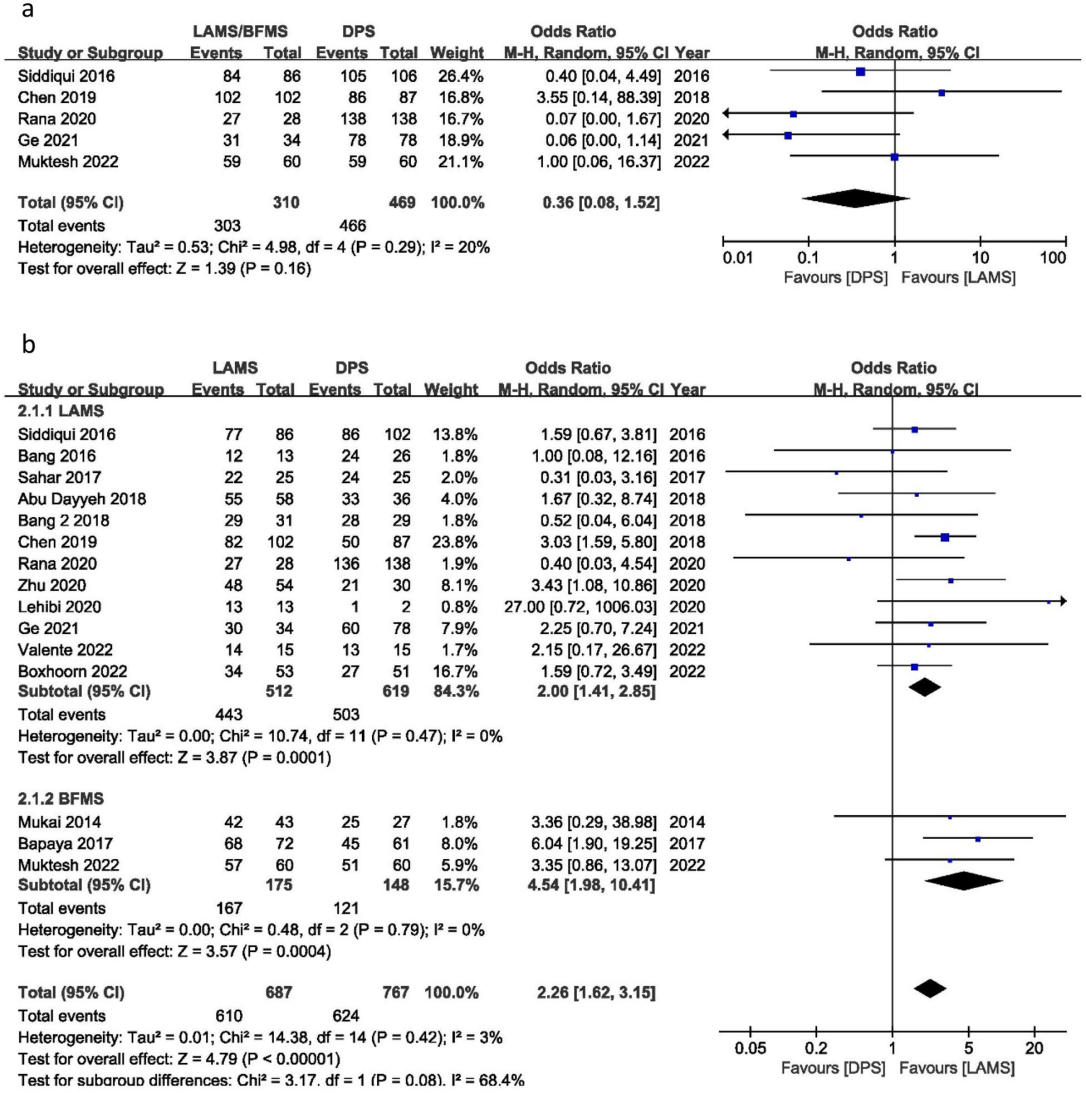

**Figure:**
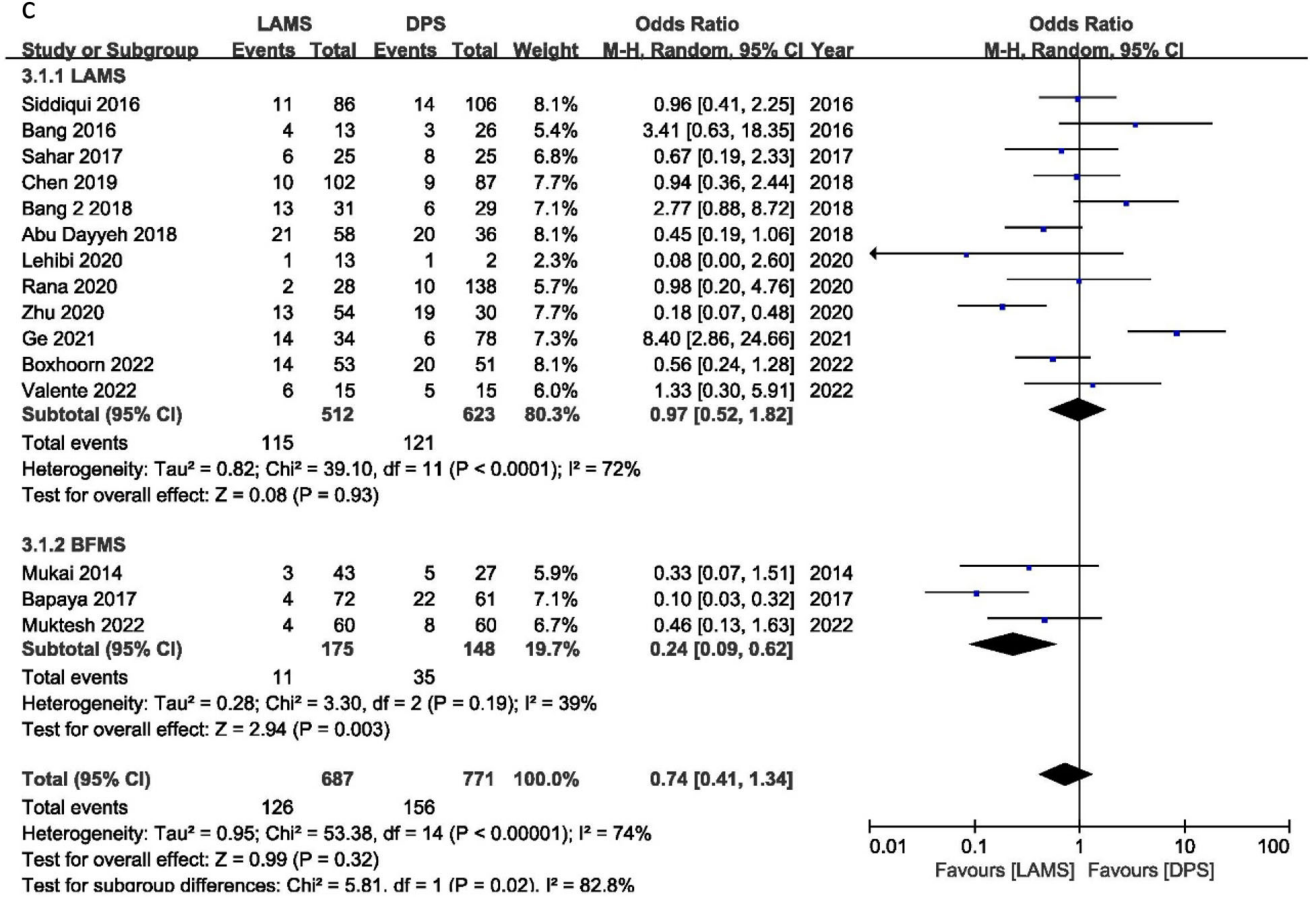

### Clinical success

Clinical success OR was calculated to use all 15 studies. Clinical success OR was 2.26 (95% CI 1.62–3.15), *I*^2^ = 3%, *P* = 0.00001. There was a significant difference in clinical success between the LAMS and the DPS groups for treating WON patients. In a subgroup analysis of LAMS and BFMS, 12 studies reported an OR of 2.00 (95% CI 1.41, 2.85), *I*^2^ = 0%, *P* = 0.0001 and three studies reported an OR of 4.54 (95% CI 1.98, 10.41), *I*^2^ = 0%, *P* = 0.0004, respectively. There was no significant difference in TS between the BFMS and the LAMS (Figure 2b).

## Secondary outcomes

### Adverse events

The OR for overall AEs was 0.74 (95% CI 0.41, 1.34), *I*^2^ = 74%, *P* = 0.32. LAMS and DPS had the same number of AEs. In the subgroup, LAMS had an OR of 0.97 (95% CI 0.52, 1.82) *I*^2^ = 72%, *P* = 0.93, while BFMS had an OR of 0.24 (95% CI 0.09, 0.62) *I*^2^ = 39%, *P* = 0.003. Subgroup results show fewer AEs for BFMS. Only three trials were published for BFMS, and Bapaya et al. considerably affected the final result of the subgroup analysis (Figure 2c).

### Bleeding

**Figure 3.**
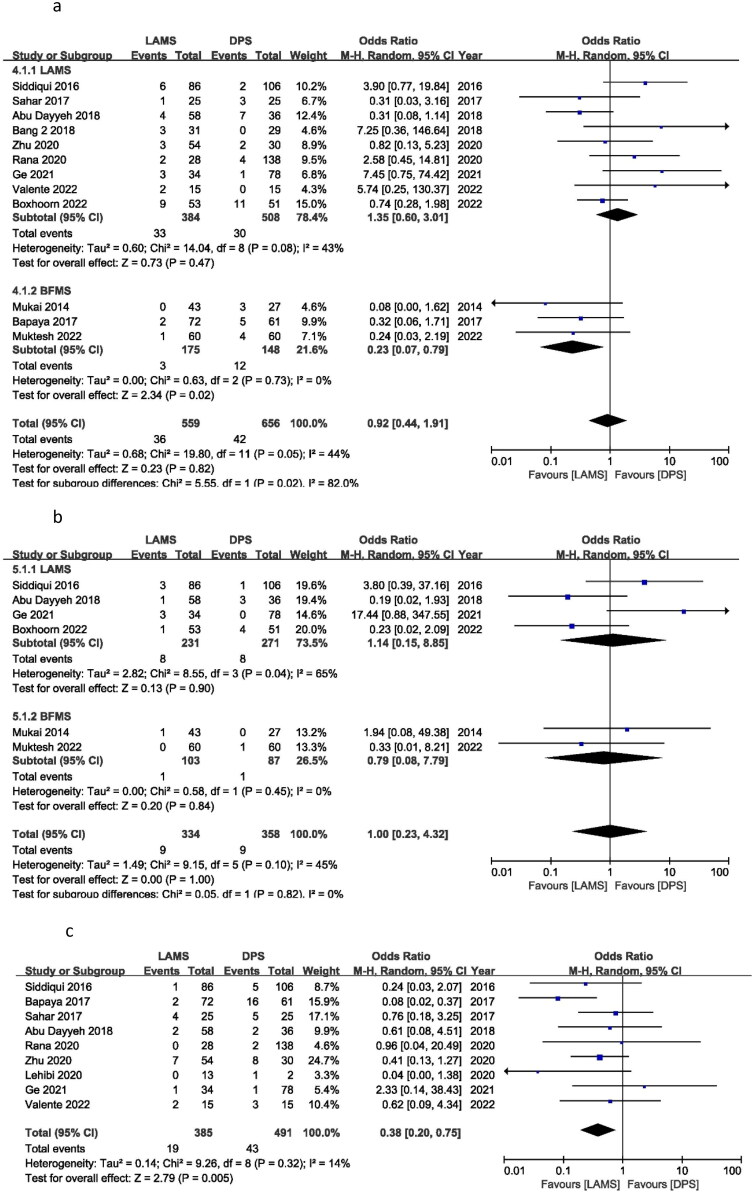
Forest plot for (a) Bleeding, (b) Perforation and (c) infection.

For PFC, bleeding is the most frequent AE. Twelve studies reported bleeding, with an OR of 0.92 (95% CI 0.44, 1.91) *I*^2^ = 44%, *P* = 0.82. For bleeding cases, there is no significant difference between DPS and LAMS. According to subgroup analysis, the ORs were 1.35 (95% CI 0.60, 3.01), *I*^2^ = 43%, *P* = 0.47 and 0.23 (95% CI 0.07, 0.79), *I*^2^ = 0%, *P* = 0.02 for LAMS and BFMS, respectively. LAMS has higher bleeding cases than BFMS. But maybe this is related to the drainage’s position, age and duration (Figure 3a).

## Perforation

Six trials reported perforation incidents with OR = 1.00 (95% CI: 0.23, 4.32), *I*^2^ = 45%, and *P* = 1.00. LAMS and DPS perforation cases were similar. BFMS had an OR of 0.79 (95% CI 0.08, 7.79), *I*^2^ = 0%, *P* = 0.84, while LAMS had an OR of 1.14 (95% CI 0.15, 8.85), *I*^2^ = 65% and *P* = 0.90. LAMS and BFMS have similar perforation rates (Figure 3b).

## Infection

Nine studies reported infection events and it showed an OR of 0.38 (95% CI 0.20, 0.75), *I*^2^ = 14%, *P* = 0.005. Infection cases are significantly lower in the LAMS group compared with BFMS. No subgroup analysis was performed between the LAMS and the BFMS (Figure 3c).

## Mortality

**Figure 4.**
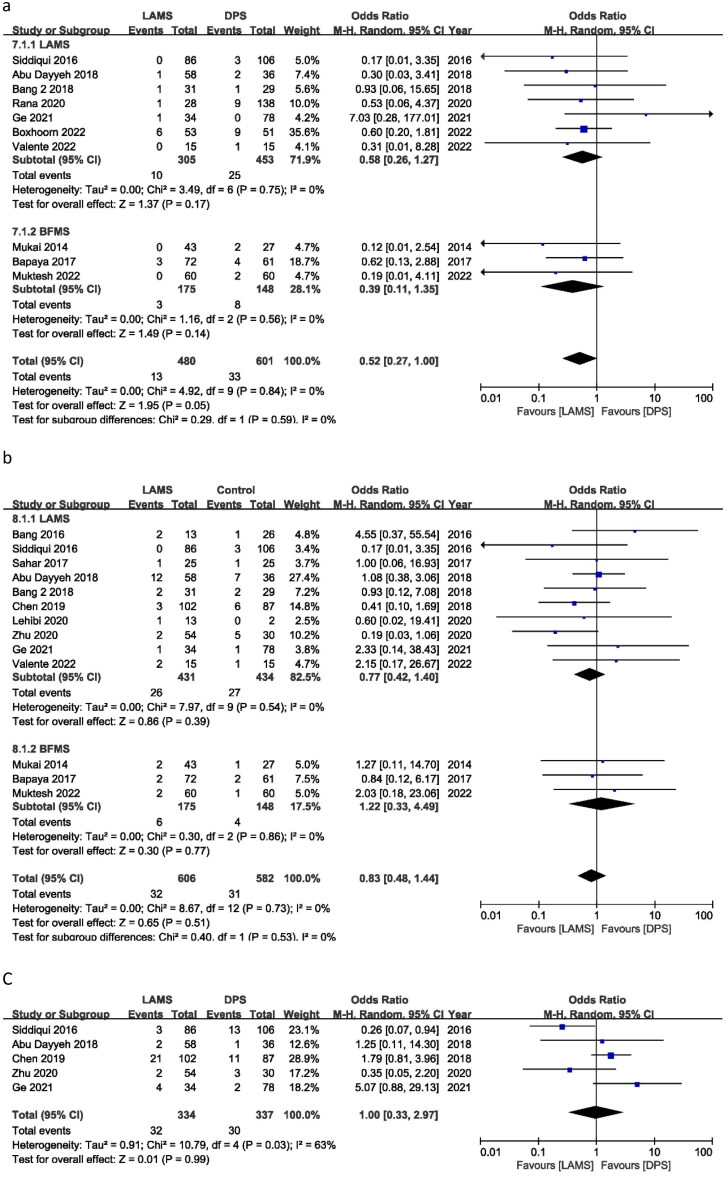
Forest plot for (a) Mortality, (b) Stent migration and (c) Stent occlusion.

During follow-up, 10 studies reported patient deaths. The OR was 0.52 (95% CI: 0.27, 1.00), *I*^2^ = 0%, and *P* = 0.05. The DPS group resulted in more patient deaths than the LAMS group. In contrast, BFMS showed an OR of 0.39 (95% CI 0.11, 1.35), *I*^2^ = 0% and *P* = 0.14, and LAMS showed an OR of 0.58 (95% CI 0.26, 1.27), *I*^2^ = 0% and *P* = 0.17. In subgroup analysis, there was no mortality difference between BFMS and LAMS (Figure 4a).

## Stent migration

Stent migration occurrences were recorded in thirteen studies. Stent migration did not differ between the LAMS and the DPS groups according to the OR of 0.83 (95% CI 0.48, 1.44), *I*^2^ = 0%, and *P* = 0.51. In subgroup analysis, BFMS produced an OR of 1.22 (95% CI 0.33, 4.49), *I*^2^ = 0%, and *P* = 0.77, while LAMS showed an OR of 0.77 (95% CI 0.42, 1.40), *I*^2^ = 0%, and *P* = 0.39. Stent migration incidents between BFMS and LAMS were the same (Figure 4b).

## Stent occlusion

Stent occlusion cases were recorded in five studies. The OR for stent occlusion was 1.00 (95% CI 0.33, 2.97), *I*^2^ = 63%, and *P* = 0.99. Between the LAMS and the DPS groups, there were no discernible differences in the number of stent occlusion occurrences (Figure 4c).

## Recurrence of WON

**Figure 5.**
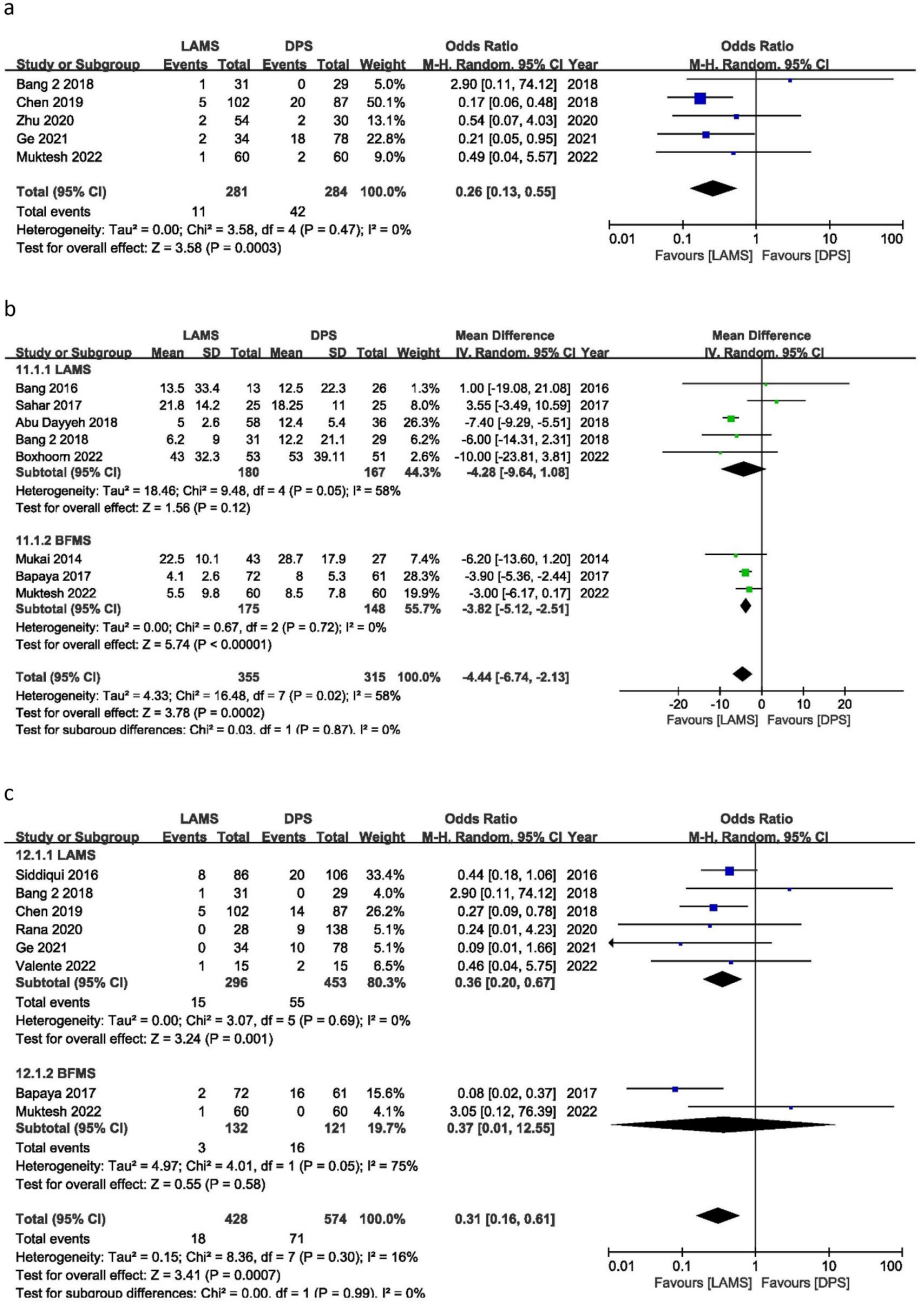
Forest plot for (a) Recurrence, (b) Hospital stay and (c) Surgical necrosectomy.

Five studies reported recurrences of WON events producing OR of 0.26 (95% CI 0.13, 0.55), *I*^2^ = 0% and *P* = 0.0003. OR indicated a significant difference in recurrences between the LAMS and the DPS groups in favour of LAMS (Figure 5a).

## Hospital stay

Eight trials documented stent-related hospital stays. The pooled mean difference between LAMS and DPS was −5.11 days (CI: –7.09, −3.14), *I*^2^ = 0% and *P* = 0.00001. The LAMS subgroup had a shorter hospital stay. In the subgroup, LAMS exhibited a mean difference of −7.31 days (95% CI −9.13, −5.49), *I*^2^ = 0% and *P* = 0.00001; BFMS showed a mean difference of −3.82 days (95% CI − = 0.00001; BFM*I*^2^ = 0% and *P* = 0.00001. The LAMS and BFMS hospitalizations were very different. LAMS demonstrated shorter hospital stays than DPS and BFMS (Figure 5b).

## Surgical necrosectomy

In eight trials, surgical necrosectomy was required; the OR was 0.31 (95% CI 0.16, 0.61), *I*^2^ = 16% and *P* = 0.0007. LAMS and DPS have different rates of necrosectomy. This is because most of patients in LAMS can perform endoscopic necrosectomies and there was no need for additional surgical necrosectomy whereas in DPS group unable to perform endoscopic necrosectomy, so they need surgical necrosectomy. That is why fewer patients in the LAMS group need surgical necrosectomy and OR was significantly in the favour of the LAMS group. Subgroup analyses of the LAMS and BFMS studies revealed substantially different ORs of 0.36 (95% CI 0.20, 0.67) *I*^2^ = 0% and *P* = 0.001 and 0.37 (95% CI 0.01, 12.55) with 75% *I*^2^ and *P* = 0.58. LAMS has fewer necrosectomies than BFMS and DPS (Figure 5c).

## Mean number of procedures for PFC drainage

Ten investigations compared LAMS and DPS drainage processes. Seven studies reported a pooled mean difference of −0.41 (95% CI: −0.70, −0.12), *I*^2^ = 56% and *P* = 0.005. In subgroup analysis, the BFMS showed a pooled mean difference of −1.09, −0.30), *I*^2^ = 75% and *P* = 0.58, whereas the LAMS showed a mean difference of −0.25 (95% CI-0.51, 0.01), *I*^2^ = 20% and *P* = 0.06. LAMS has fewer drainage interventions than DPS and BFMS. Regarding the BFMS, data from only two studies were used, and the findings of Bapaya et al. have a higher influence on the outcome. When drawing any conclusions based on the findings of this investigation, need to keep this impact in mind (Fig. S1a).

## Mean procedure time

Five studies reported the procedure’s average time for the implantation of the stents. The pooled mean difference was statistically significant in favour of the LAMS, −26.16 min (95% CI −28.68, −23.65) *I*^2^ = 0% and *P* < 0.00001. The LAMS group took less time than the DPS group to complete the stent implantation (Fig. S1b).

## Discussion

This systematic review and meta-analysis compared the safety and efficacy of EUS-guided drainage using the LAMS/BFMS and the DPS for WON patients. The LAMS has a higher clinical success rate than the DPS, similar AEs, a shorter hospital stay, shorter procedure duration and fewer procedure sessions. Only two of the 15 studies were randomized controlled trials (RCTs). Thirteen were observational. This is the first meta-analysis comparing LAMS and DPS for WON drainage. It also compares BFMS and LAMS subgroups.

EUS-guided PFC drainage is the standard treatment for WON. In a RCT of patients with infected necrotizing pancreatitis, endoscopic necrosectomy was associated with reduced morbidity compared with surgical necrosectomy [4]. A systematic review and meta-analysis of nine studies of WON comparing the outcomes of the LAMS and the DPS groups suggested that LAMS showed higher clinical success and comparable AEs. But they included two abstract studies in the meta-analysis [35]. In contrast, we had our meta-analysis’s 15 most recent full-text articles and independently compared LAMS and BFMS outcomes in a subgroup analysis. Studies comparing BFMS and LAMS for WON treatment have shown that the two methods produce similar results with fewer AE [36,37]. Our subgroup analysis also showed the same outcome results for the LAMS and the DPS, but only three studies reporting the outcome of the BFMS compared with the DPS were included in our analysis. Such small data is not adequate for final results. Another meta-analysis by Mohan et al. [38] The LAMS and the DPS showed 88.5% and 88.1% clinical success, respectively. According to the author, there was no distinction between the LAMS and the DPS regarding the clinical success or AEs when treating WON. However, our analysis revealed that the LAMS had superior clinical outcomes, shorter hospital stays, and similar AEs. In contrast to this meta-analysis, we only included WON studies that compared LAMS and DPS outcomes. We did not include any studies that used a single control group. Recent research made use of advanced and improved LAMS was included. Maybe these are the aspects that make our analysis different from other studies.

A number of meta-analyses have demonstrated the LAMS’s superiority over the PFC’s DPS. A recent meta-analysis comparing 13 studies reporting outcomes of the LAMS and DPS for PFC suggested that both groups have higher technical and clinical success, but LAMS showed lower AEs. Compared with our study, this meta-analysis included patients with pancreatic fluids [39]. Another case series and meta-analysis by Li et al. [40] also reported that for PFC, LAMS showed preferable clinical success and AEs. A meta-analysis of eleven studies comparing the data of 688 patients with PFC through the LAMS and the DPS indicated that the LAMS showed better efficacy in managing PFC [41]. Another meta-analysis comparing 15 studies with PFC suggests that LAMS has higher clinical success, fewer recurrences and fewer additional interventions [42]. Our analysis also confirmed same WON’s results. First, we compared LAMS and BFMS separately. In overall analysis, LAMS and BFMS were regarded the same kind, however in subgroup analysis, they were separated. Many studies have shown that the LAMS is safer and more effective for bile collection for biliary and gallbladder illnesses [43–46]. Single-arm studies also showed LAMS are safe and effective. A recent study found no difference between 10, 15 and 20 mm LAMS for PFC [47]. Another study also indicated that endoscopic drainage of WON patients is effective and less invasive than surgery [48].

LAMS’s direct necrosectomy channel is its main advantage over DPS. The larger LAMS diameter allows for faster and more spontaneous direct draining into the stomach or duodenum, allowing necrosectomy without extra intervention. This improves patient comfort, speeds recovery, and shortens hospital stays. The improved LAMS has a simple and quick deployment system. Necrosectomy demands more time and effort for DPS and need to perform surgical necrosectomy. Only four studies reported data about the additional percutaneous drainage method but they did not explain any impact of this additional drainage method on the final outcome. To elaborate this impact more studies are needed more focussing this aspect. Our meta-analysis also showed that OR for surgical necrosectomy was in the favour of the LAMS that mean less patients in the LAMS group need surgical necrosectomy. Only two RCTs comparing WON treatments have been described. Surprisingly, the LAMS did not exhibit the expected clinical success advantage over the DPS. Both RCTs found different AEs. In meta-analyses, LAMS did better than DPS. Most of the studies included in meta-analyses are observational studies with various follow-up lengths, PFC sizes, stent counts and recording methods. RCT studies must follow a predetermined protocol to track outcomes and endpoints. More RCTs are needed to provide more precise and accurate data.

Stent related AEs were almost same for both the LAMS and DPS. The large lumen of LAMS made it possible to rapidly drain the liquid component but left the solid component in the cavity, where it eventually caused stent obstruction; and the larger number of side holes in PS meant that the stent could not be completely blocked. Stent obstruction can occur when a large quantity of solid debris is discharged through a large lumen LAMS and some of the solid components accumulate in the stent. So, PS placement in LAMS has been suggested as a way to reduce complications caused by stents. According to a study by Brimhall et al. [49], postoperative complications of LAMS were found to account for 24.7% of cases, with stent-related haemorrhage accounting for 15.5% of these cases and pseudoaneurysm haemorrhage accounting for ∼8.2% of these cases. Further prospective studies are required to accurately characterize patients who have LAMS-associated bleeding. But it is possible that the presence of pseudoaneurysms could raise the risk of bleeding caused by LAMS.

Most of the included articles are observational studies, not RCTs. More RCTs are needed to assess WON treatments’ safety and efficacy. Different research defines therapy effectiveness differently, which could influence our real outcome and primary purpose. Only three BFMS-reporting trials were considered for subgroup analysis. These results do not prove the BFMS. More research is needed to compare the LAMS and BFMS’ efficacy and safety. The included studies have varying follow-up lengths. We didn’t assess how long researchers followed participants. Specific experiments used hydrogen peroxide irrigation, nasocystic drainage, percutaneous drainage and antibiotics, which may have altered results. So, more studies focussing these impacts are needed. Methodological restrictions must be considered.

## Conclusion

When comparing the LAMS to the DPS for the treatment of WON, this meta-analysis of fifteen studies found that the LAMS is associated with superior clinical outcomes, shorter hospital stays and procedure times, fewer interventions, and equivalent AEs. Results from subgroup analysis showed no difference between the BFMS and LAMS.

## Approval and consent to publish

All authors have approved to this submission to your esteemed journal. Its publication is also approved tacitly by the responsible authorities where the work was carried out.

## Informed consent statement

Not applicable.

## Institutional review board statement

Not applicable.

## Supplementary Material

Supplemental Material

## Data Availability

The supporting data of this study is available in supplementary materials and more information can be found on request from the corresponding author (YJF), upon reasonable request.
