# Supplementary material for: Safety and efficacy of lumen-apposing metal stents and double-pigtail plastic stents for endoscopic ultrasound-guided drainage of walled-off necrosis; a systematic review and meta-analysis
Source: Ann Med. 2023 Feb 13;55(1):578–91. doi: 10.1080/07853890.2022.2164048 (PMC9930761; doi:10.1080/07853890.2022.2164048)

**Forest plots a)** Mean number of procedures b) Mean procedure time.

**a**

**
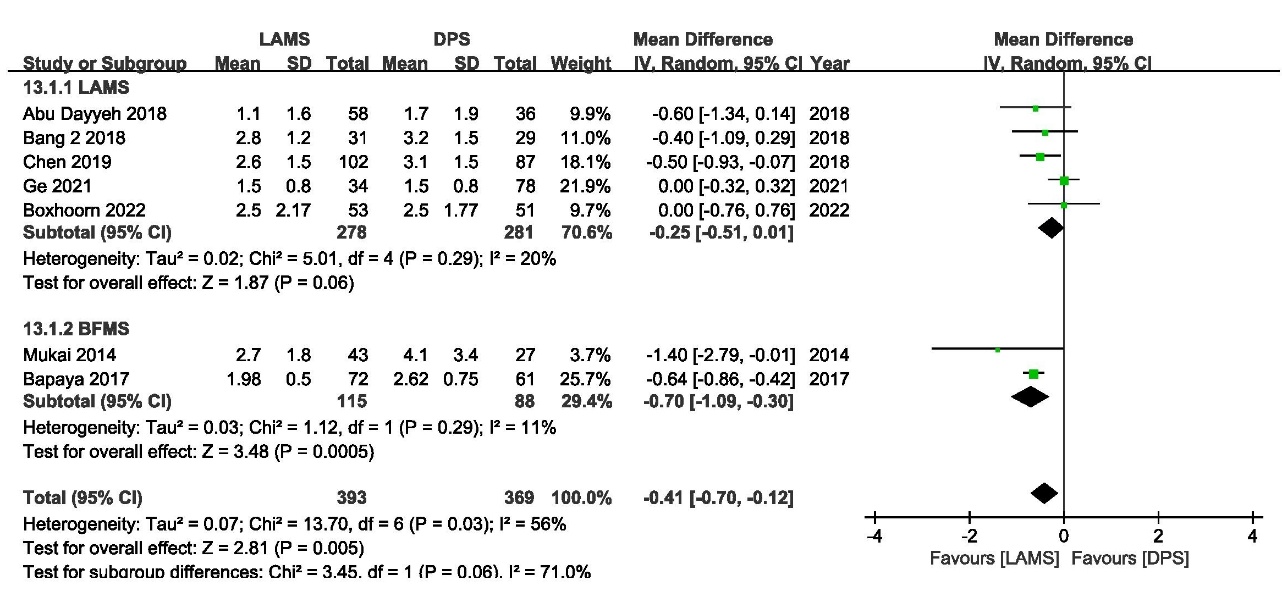
**

**b**

**
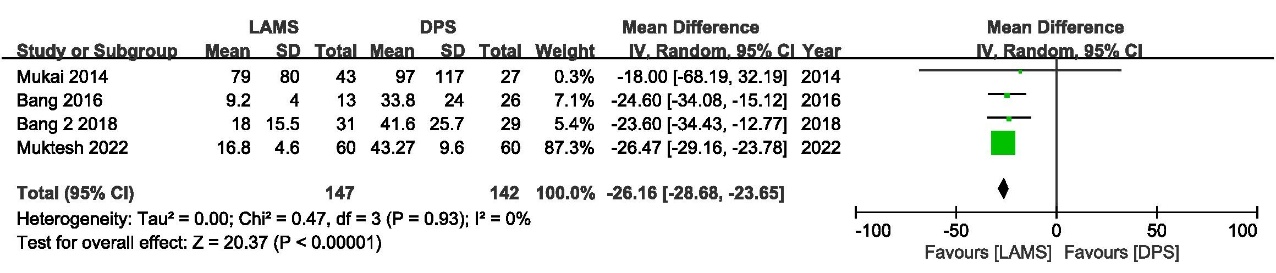
**

**Table 1** **Risk of Bias assessment using the Cochran Risk of Bias Tool (ROBINS-1) for Studies;**

| Study | Bias due to confounding | Bias in selection of participants into the study | Bias in measurement of interventions | Bias due to deviation from intended interventions | Bias due to missing data | Bias in measurement of outcomes | Bias in selection of the reported result | Overall bias |
| --- | --- | --- | --- | --- | --- | --- | --- | --- |
| Mukai et al.2014 | Moderate | Low | Low | Low | Low | Low | Low | Moderate |
| Siddiqui et al. 2016 | Moderate | Low | Low | Low | Low | Low | Low | Moderate |
| Bang et al 2016 | Moderate | Low | Low | Low | Low | Low | Low | Moderate |
| Abu Dayyeh et al 2018 | Moderate | Low | Low | Low | Low | Low | Low | Moderate |
| Sahar et al. 2017 | Moderate | Low | Low | Low | Low | Low | Low | Moderate |
| Bapaya et al. 2017 | Moderate | Low | Low | Low | Low | Low | Low | Moderate |
| Bang 2 et al. 2018 | Low | Low | Low | Low | Low | Low | Low | Low |
| Chen et al. 2019 | Moderate | Low | Low | Low | Low | Low | Low | Moderate |
| Rana et al. 2020 | Moderate | Low | Low | Low | Low | Low | Low | Moderate |
| Zhu et al. 2020 | Moderate | Low | Low | Low | Low | Low | Low | Moderate |
| Lehibi et al. 2020 | Moderate | Low | Low | Low | Low | Low | Low | Moderate |
| Ge et al. 2021 | Moderate | Low | Low | Low | Low | Low | Low | Moderate |
| Muktesh et al.2022 | Moderate | Low | Low | Low | Low | Low | Low | Moderate |
| Boxhoorn et al. 2022 | Low | Low | Low | Low | Low | Low | Low | Low |
| Valente et al. 2022 | Moderate | Low | Low | Low | Low | Low | Low | Moderate |

**Funnel plots**

1. **Technical success**
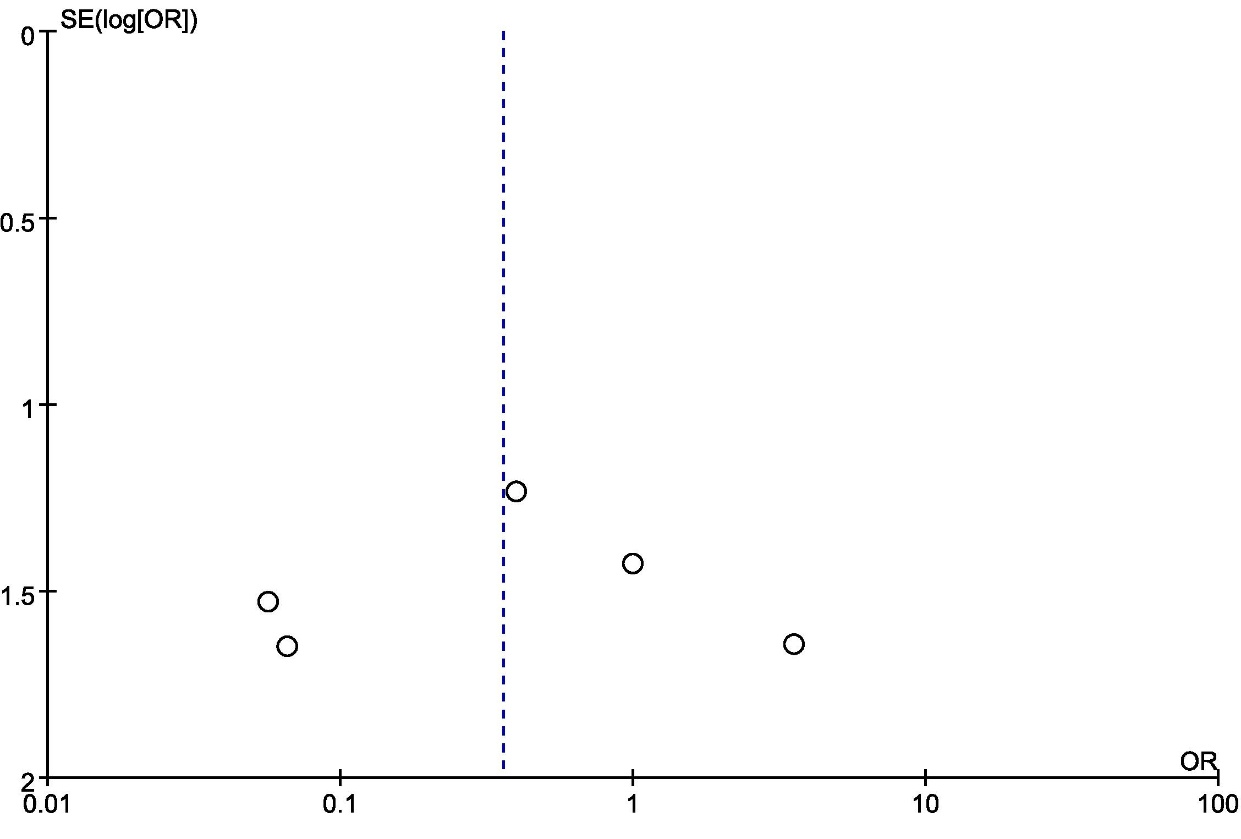

2. **Clinical success**
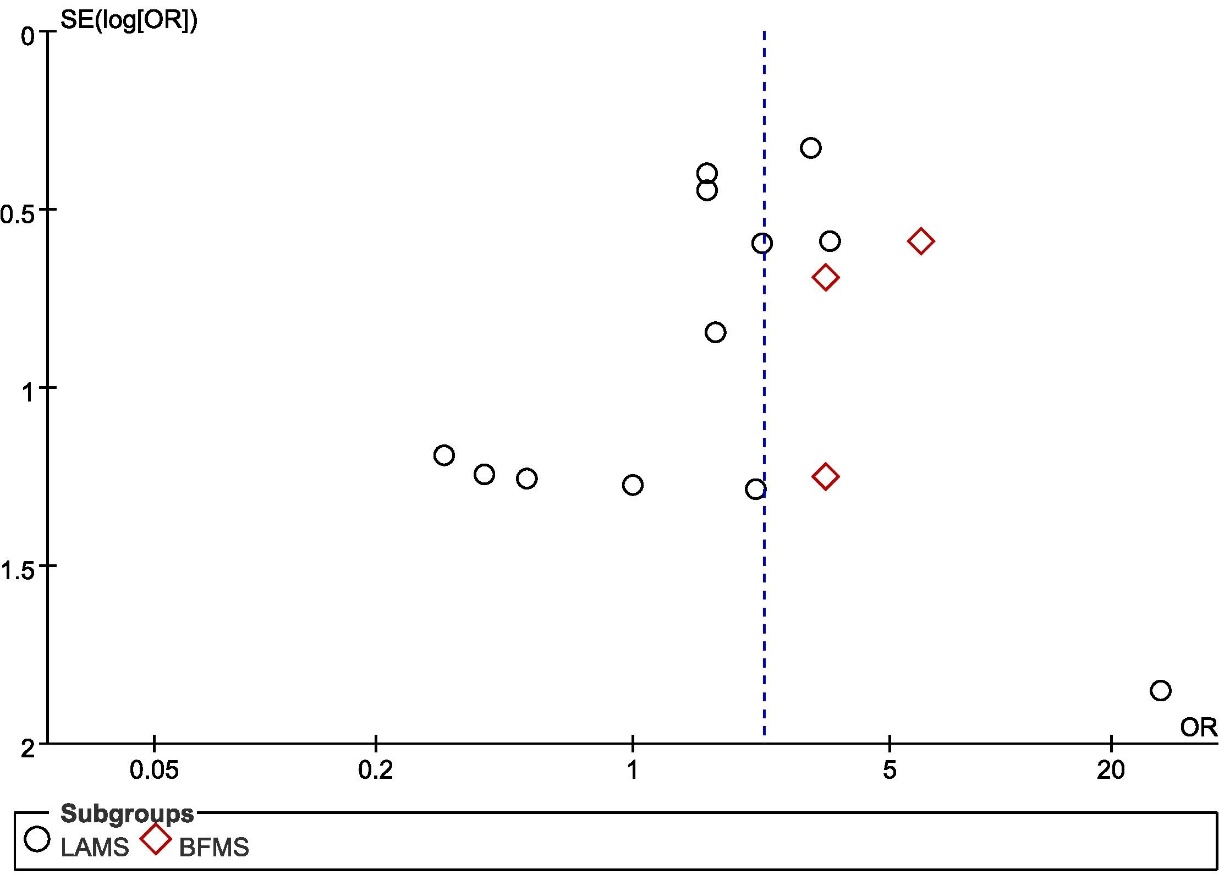

3. **Overall adverse events**
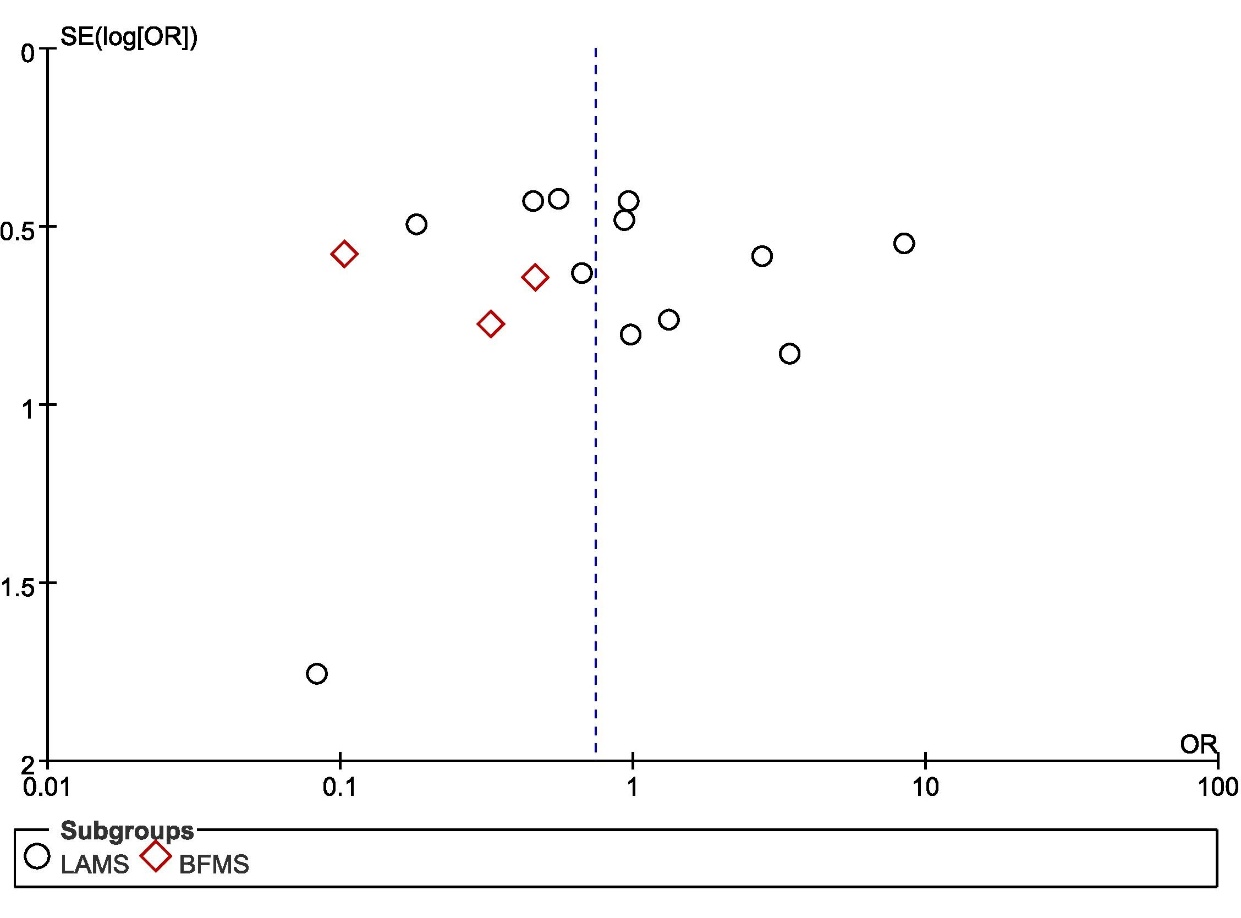

4. **Bleeding**
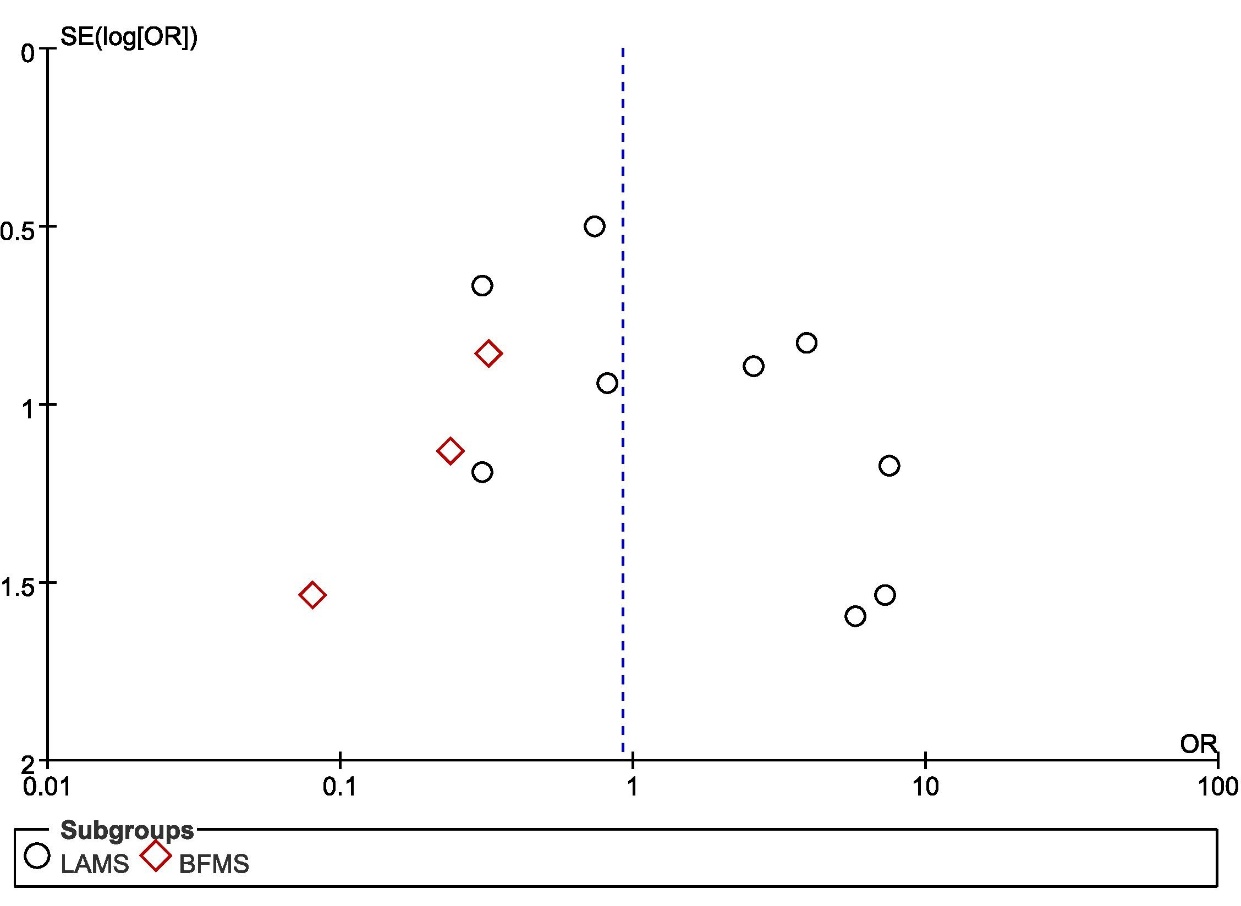

5. **Infection**
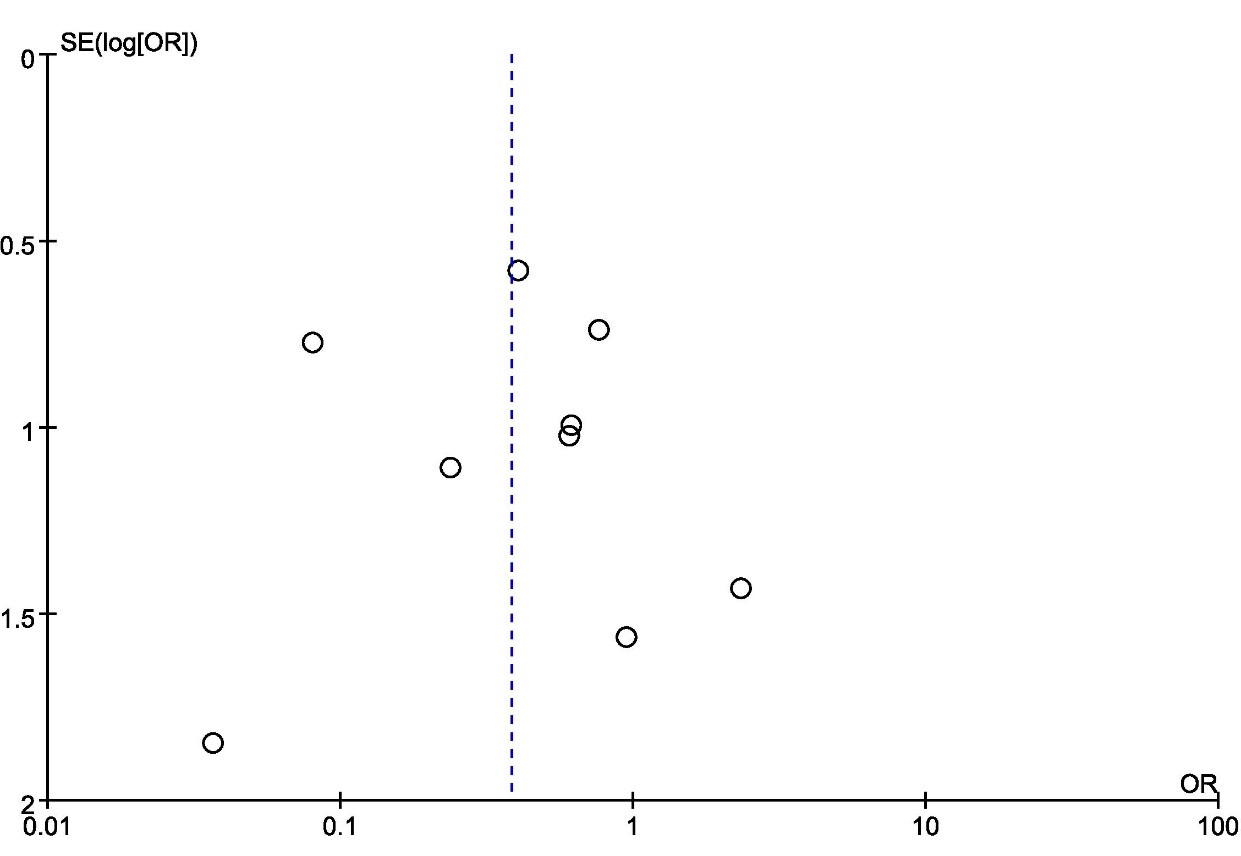

6. **Perforation**
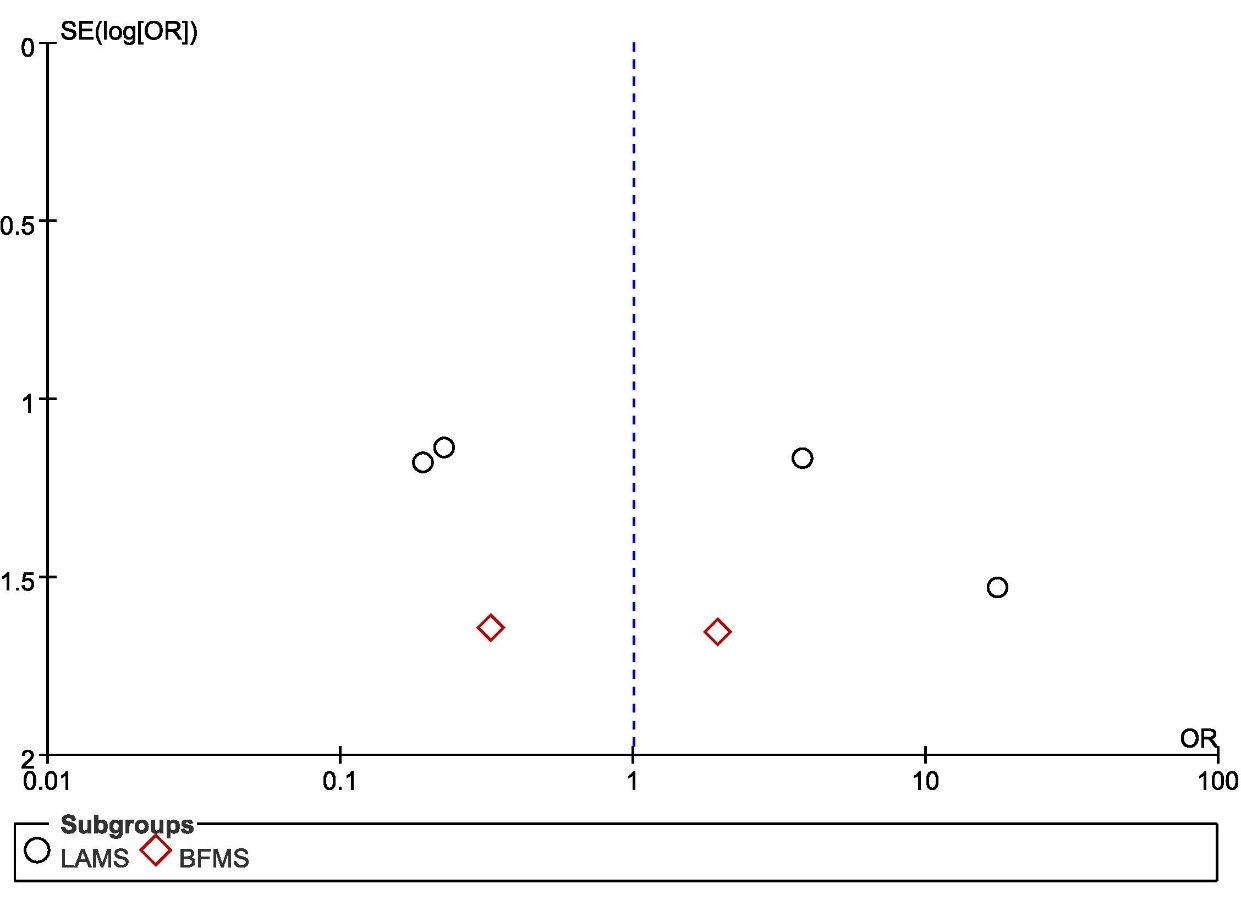

7. **Mortality**
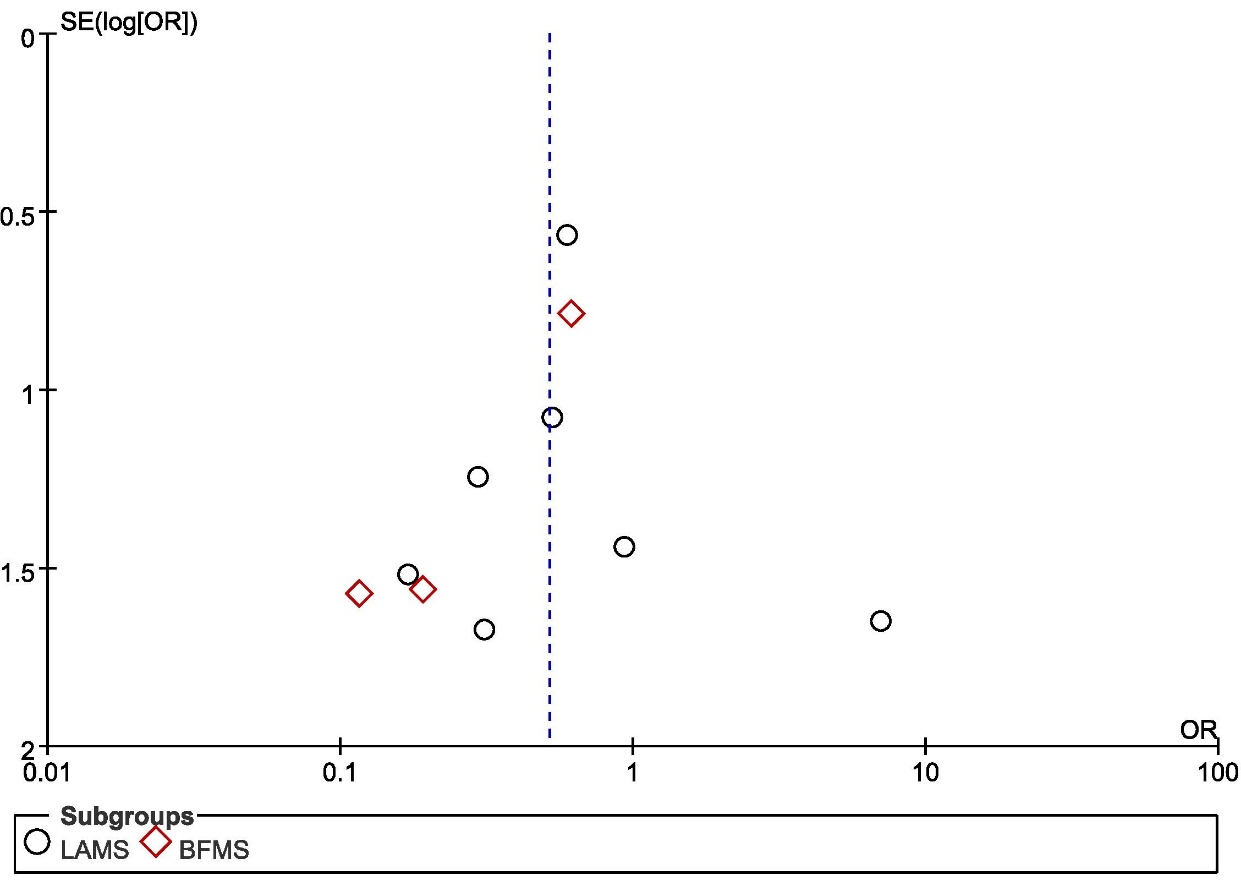

8. **Stent migration**
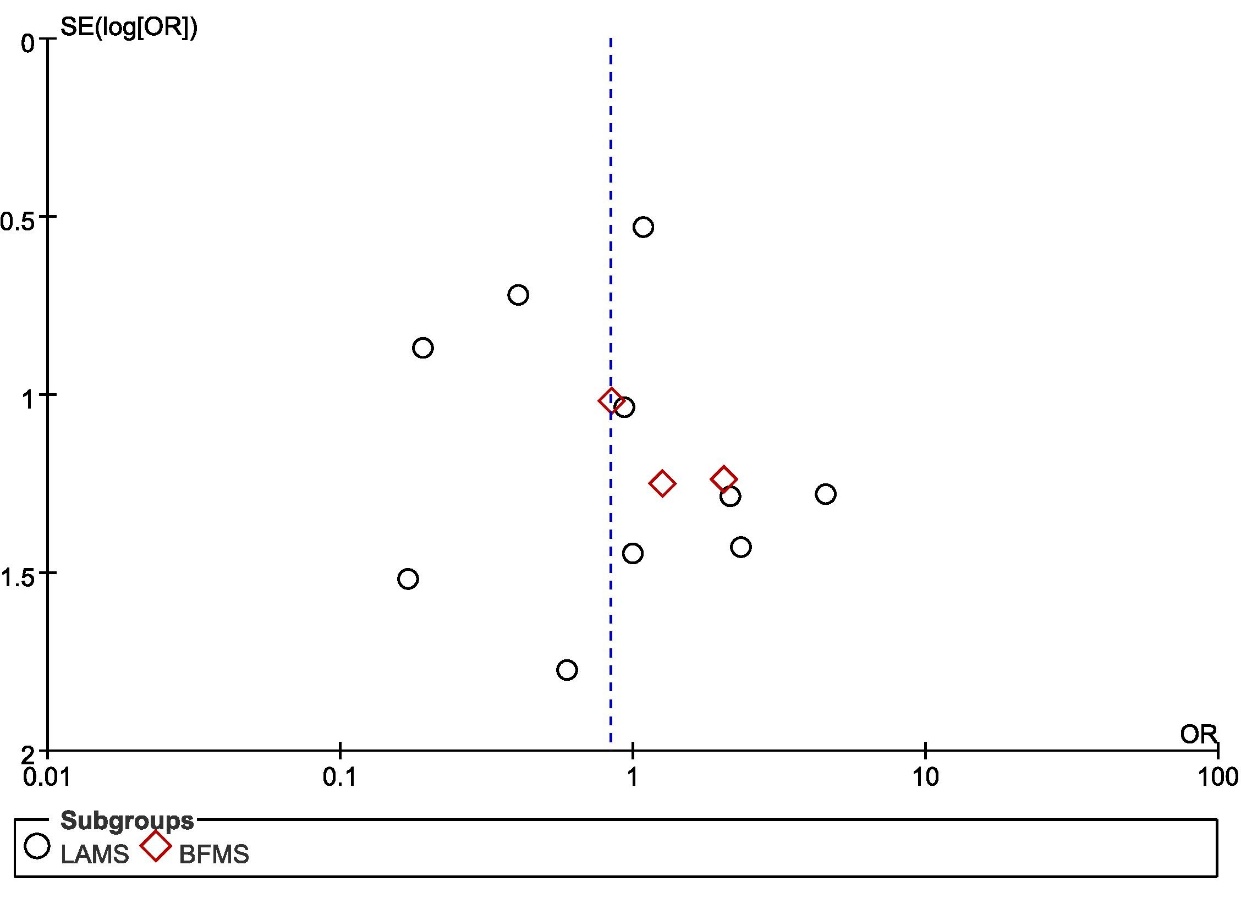

9. **Stent occlusion
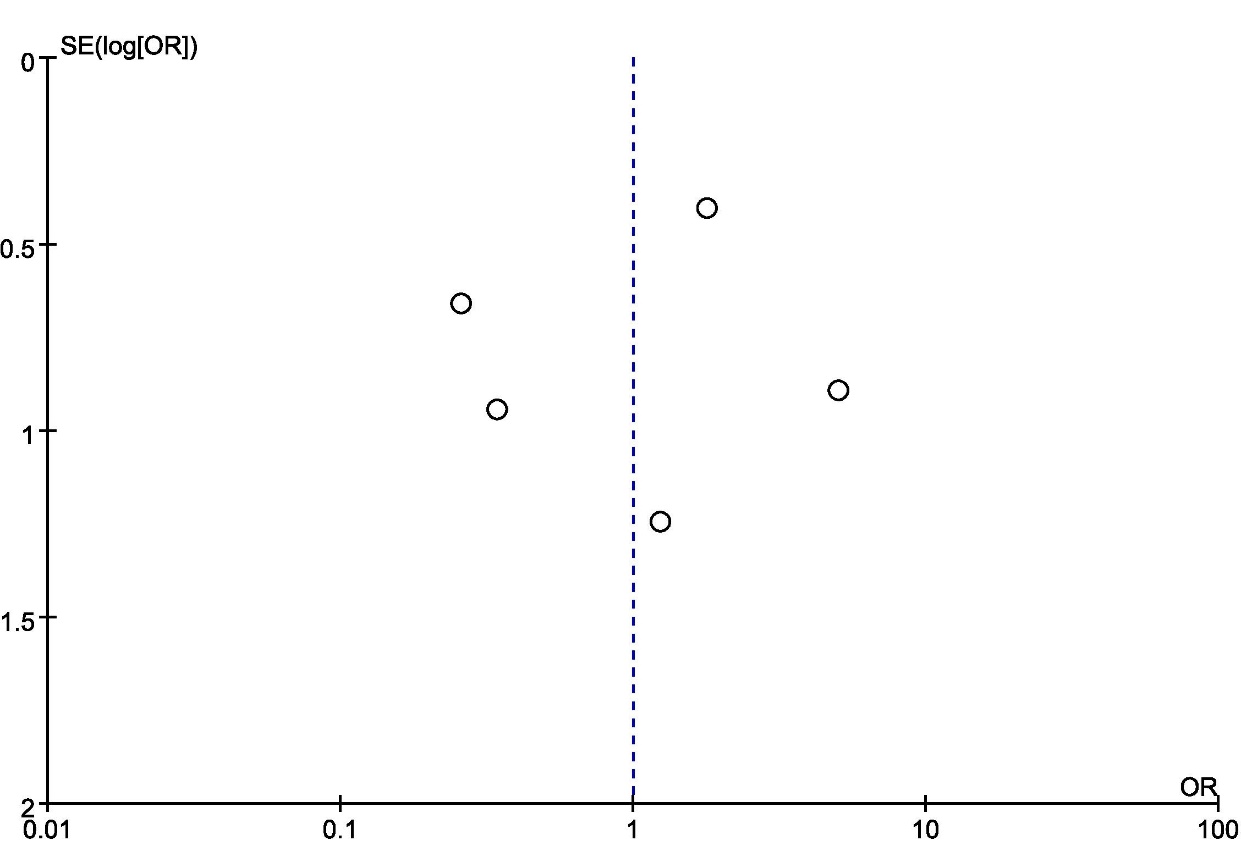
**
10. **Recurrence** of **WON**
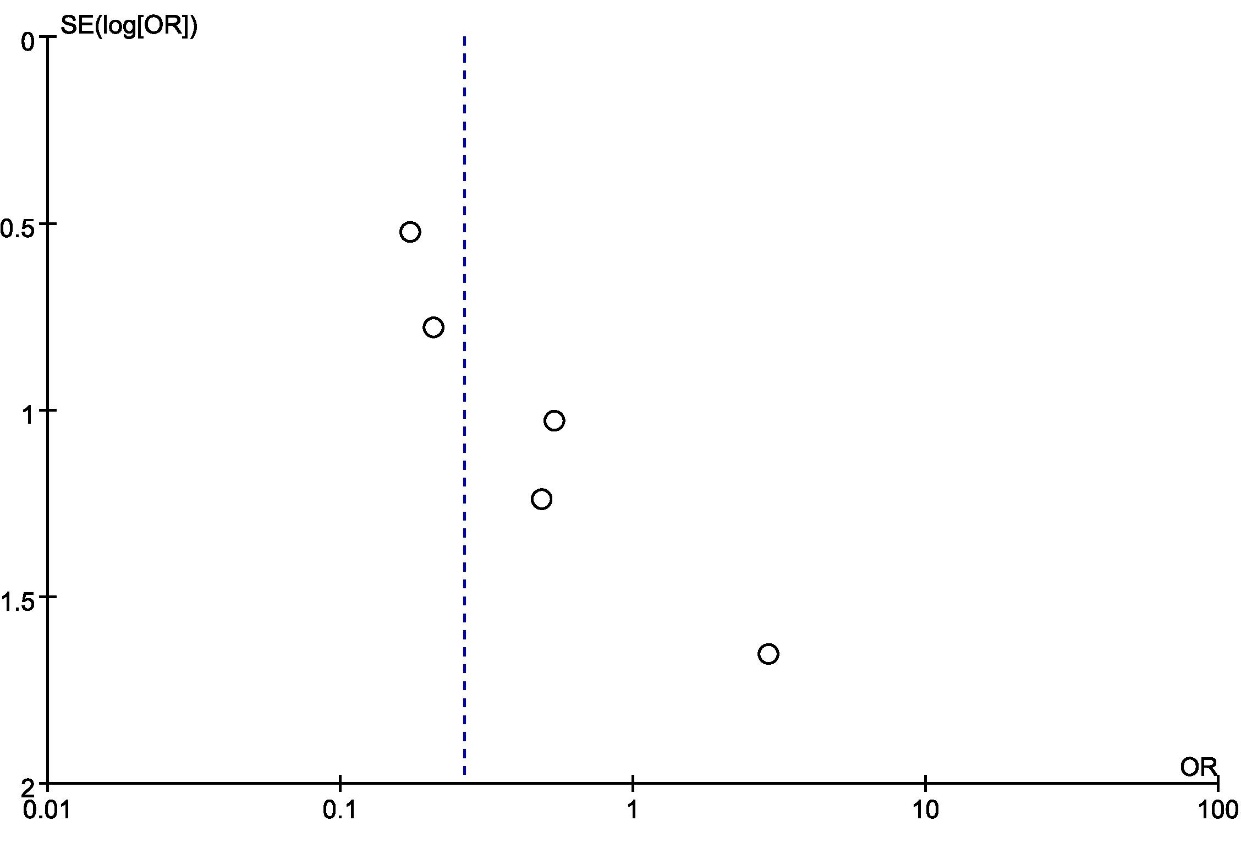

11. **Hospital stay**
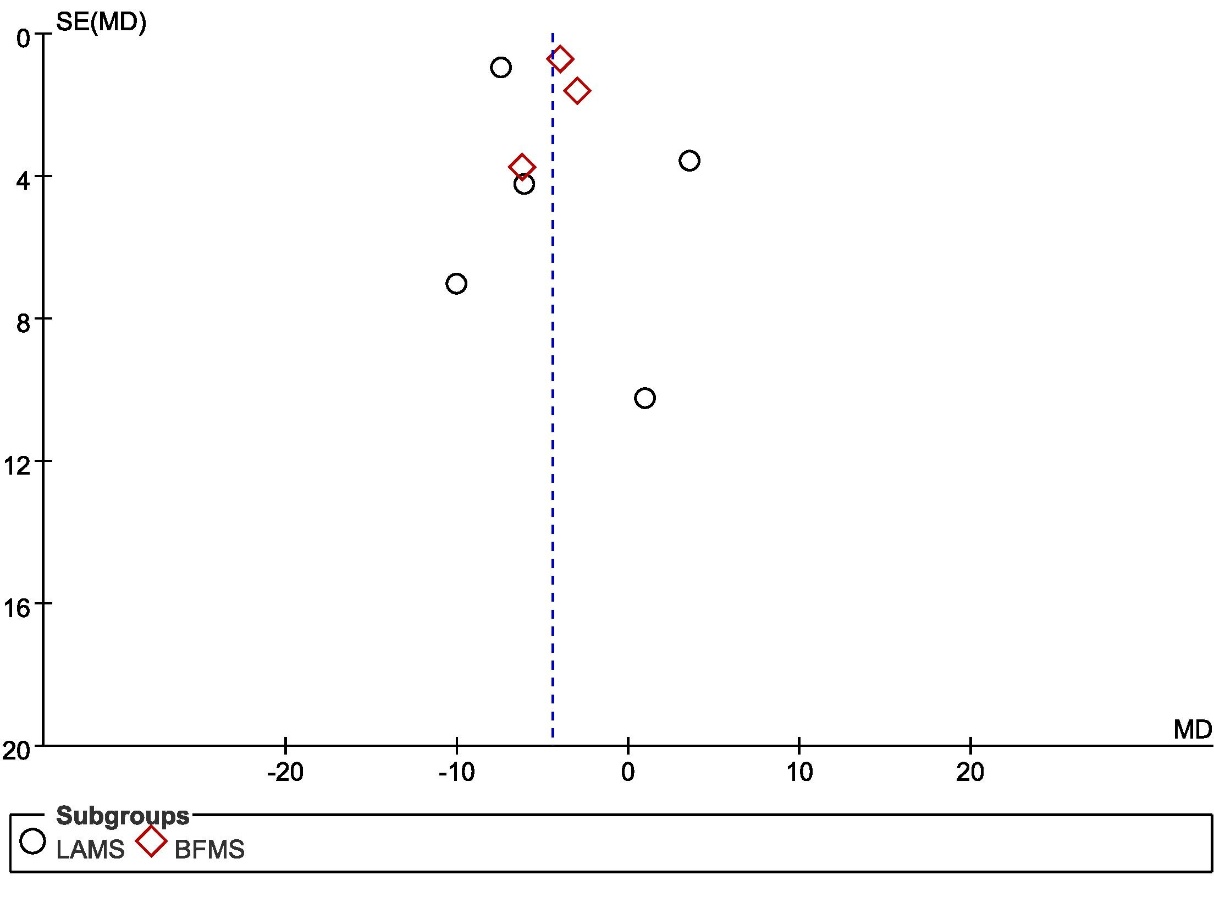

12. **Surgical necrosectomy**
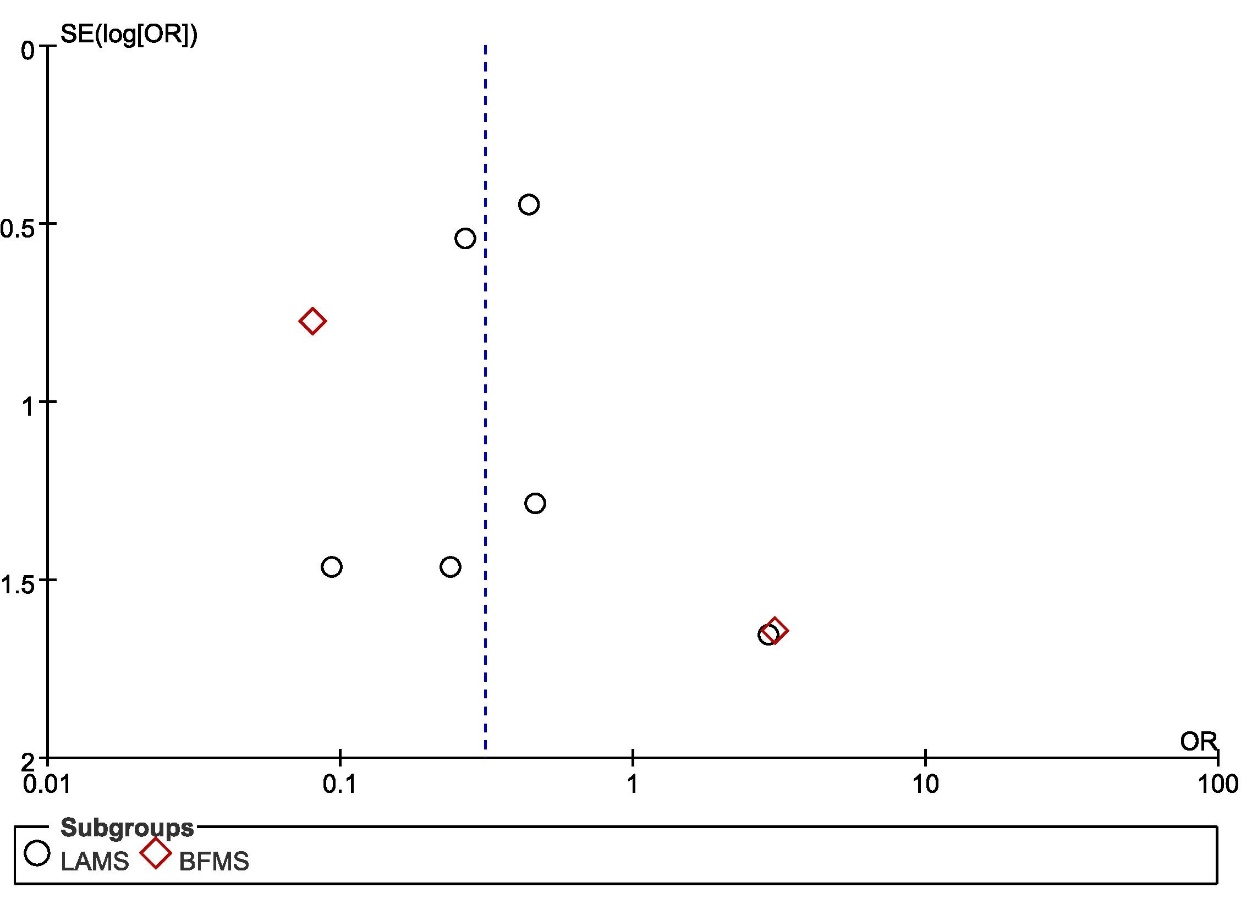

13. **Mean procedures**
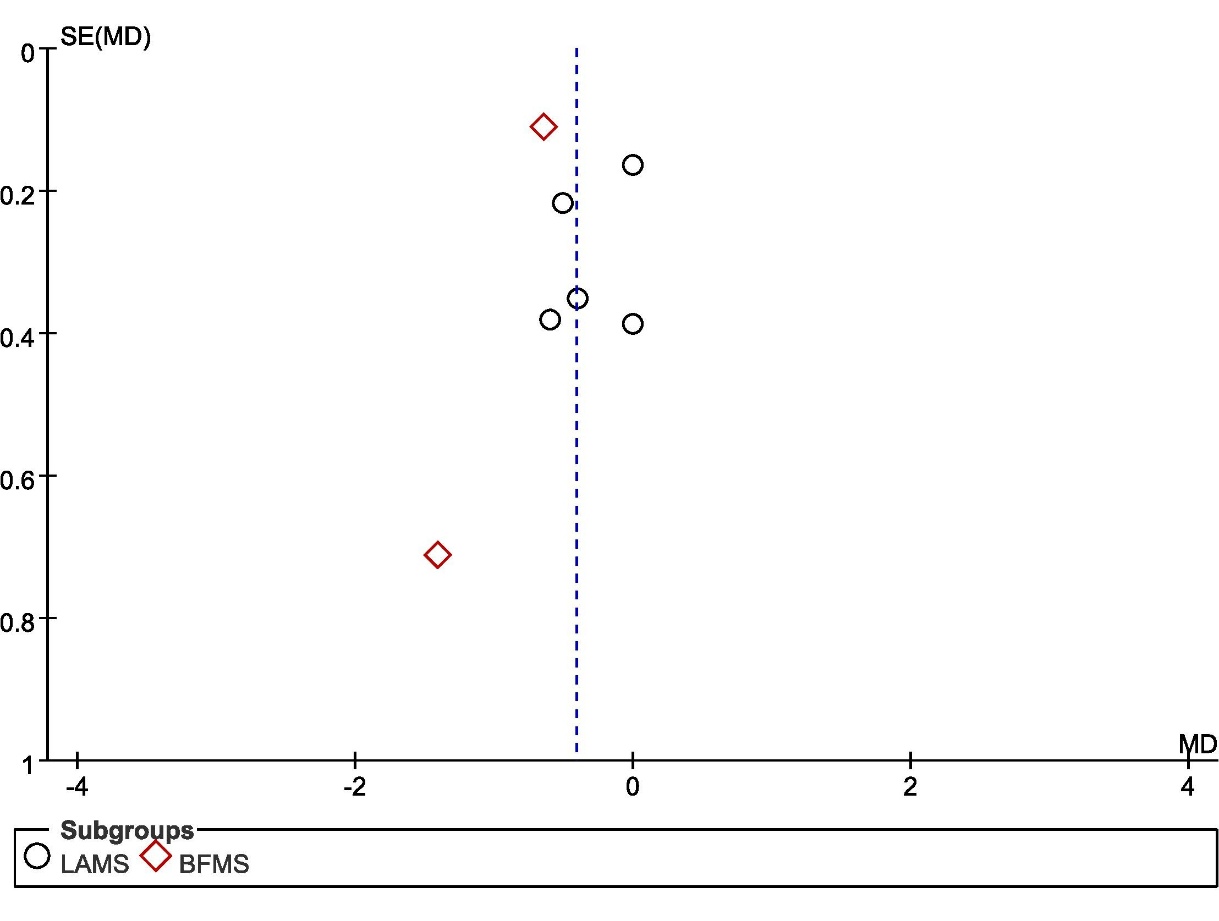

14. **Mean procedure time**
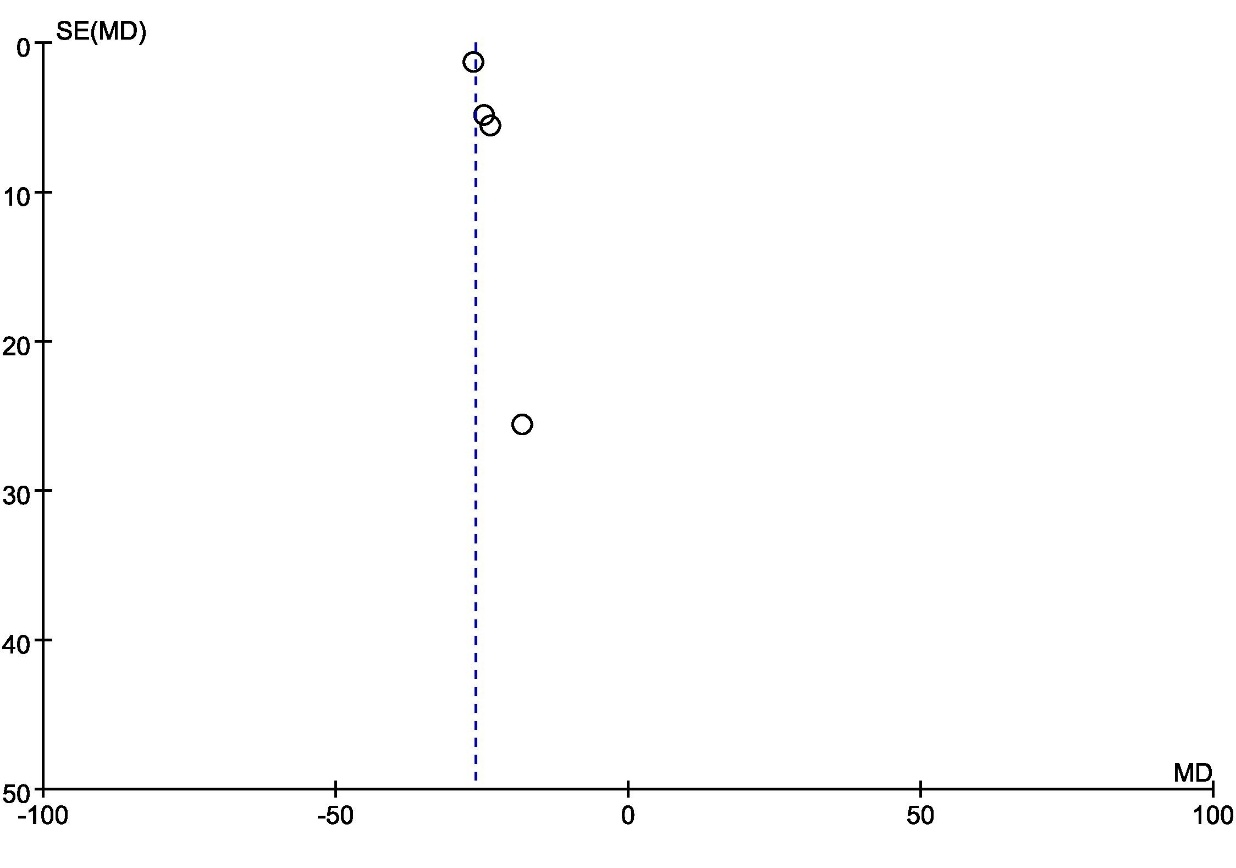

Supplement: Supplemental Material [file IANN_A_2164048_SM6057.docx]
